# Prognostic significance of diabetes mellitus in locally advanced non-small cell lung cancer

**DOI:** 10.1186/s12885-015-2012-4

**Published:** 2015-12-21

**Authors:** Hisao Imai, Kyoichi Kaira, Keita Mori, Akira Ono, Hiroaki Akamatsu, Shunichi Matsumoto, Tetsuhiko Taira, Hirotsugu Kenmotsu, Hideyuki Harada, Tateaki Naito, Haruyasu Murakami, Masahiro Endo, Takashi Nakajima, Masanobu Yamada, Toshiaki Takahashi

**Affiliations:** 1 Division of Thoracic Oncology, Shizuoka Cancer Center, 1007 Shimonagakubo, Nagaizumi-chou, Suntou-gun, Shizuoka 411-8777 Japan; 2 Clinical Trial Coordination Office, Shizuoka Cancer Center, 1007 Shimonagakubo, Nagaizumi-chou, Suntou-gun, Shizuoka 411-8777 Japan; 3 Division of Radiation Oncology, Shizuoka Cancer Center, 1007 Shimonagakubo, Nagaizumi-chou, Suntou-gun, Shizuoka 411-8777 Japan; 4 Division of Diagnostic Radiology, Shizuoka Cancer Center, 1007 Shimonagakubo, Nagaizumi-chou, Suntou-gun, Shizuoka 411-8777 Japan; 5 Division of Diagnostic Pathology, Shizuoka Cancer Center, 1007 Shimonagakubo, Nagaizumi-chou, Suntou-gun, Shizuoka 411-8777 Japan; 6 Department of Medicine and Molecular Science, Gunma University Graduate School of Medicine, 3-39-15, Showa-machi, Maebashi, Gunma 371-8511 Japan; 7 Department of Oncology Clinical Development, Gunma University Graduate School of Medicine, 3-39-15, Showa-machi, Maebashi, Gunma 371-8511 Japan

**Keywords:** Locally advanced, Non-small cell lung cancer, Prognostic factors, Diabetes mellitus, Serum factors

## Abstract

**Background:**

To investigate the prognostic significance of patient characteristics and clinical laboratory test results in locally advanced non-small cell lung cancer (NSCLC), and in particular the impact of diabetes mellitus (DM) on the survival of patients who underwent chemoradiotherapy.

**Methods:**

We retrospectively reviewed 159 patients with locally advanced NSCLC with a focus on DM and other potential prognostic factors, using the log-rank test, and univariate and multivariate analyses to assess their association with survival.

**Result:**

Five significant prognostic factors were identified in univariate analysis: stage (*p* < 0.001), DM (*p* = 0.04), hemoglobin levels (*p* = 0.003), serum albumin (*p* <0.001) and lactate dehydrogenase (LDH) levels (*p* = 0.01). Furthermore, among the factors tested using Fisher's exact test and the Wilcoxon rank sum test, gender (*p* = 0.019) and plasma glucose level (*p* <0.001) were found to have prognostic significance. Multivariate analysis showed that stage, DM, serum albumin and LDH levels were independent prognostic factors for survival (*p* = 0.007, *p* = 0.024, *p* = 0.007 and *p* = 0.005, respectively).

**Conclusions:**

The presence of DM at the time of diagnosis was identified as an independent and significant prognostic factor for predicting negative outcome in locally advanced NSCLC patients.

## Background

Lung cancer is the most common type of cancer, both worldwide and in Japan [1]. Non-small cell lung cancer (NSCLC) accounts for 80–85 % of lung cancer cases, and approximately 30 % of patients have unresectable, locally advanced disease at diagnosis [2]. Sause *et al.* reported that combining chemotherapy and radiotherapy brought a further survival benefit [3]. A recent meta-analysis concluded that concurrent chemoradiotherapy (CRT) is the most effective treatment for these patients [4], and CRT is currently the recommended standard first-line treatment for locally advanced NSCLC. Stage III NSCLC constitutes a heterogeneous group, probably due to a varying extent of nodal involvement. The median survival of patients with stage III NSCLC has recently been revised from 12 to 23.3 months based on the findings of phase III trials [5, 6].

A number of very different prognostic factors in several trials have been identified for the survival of patients with NSCLCs [7–15]. Various studies have indicated that among patients with colorectal, pancreatic, breast, or liver cancer, existing diabetes mellitus (DM) was associated with a lower chance of long-term survival [16–19]. It has also been found that good performance status (PS), disease stage, age and weight loss are strong prognostic factors in this malignancy. Although the prognostic significance of pre-existing DM in patients with lung cancer was evaluated in a number of studies [20–27], their findings were inconsistence. Three studies reported a shorter survival for patients with DM compared to those without DM [20–22]. On the other hand, several studies have shown that DM was associated with an equal or longer survival [23–27]. Thus the prognostic significance of DM in NSCLC remains uncertain.

The aim of this study was to investigate the impact of DM on the survival of patients with locally advanced NSCLC who received first-line CRT.

## Methods

### Patient population

We retrospectively reviewed the clinical records of 159 patients with clinical stage III NSCLC who were treated with concurrent CRT at the Shizuoka Cancer Center between September 2002 and December 2009. The eligibility criteria of this study were as follows: (1) histologically or cytologically proven NSCLC; (2) chemoradiotherapy naïve; (3) age ≤ 75 years; (4) Eastern Cooperative Oncology Group (ECOG) PS of 0 to 2; and (5) treated with curative thoracic radiotherapy over 50Gy concurrent with platinum doublet chemotherapy. Sixteen potential prognostic variables were chosen on the basis of previously published clinical trials [7–14]. Baseline characteristics, including the presence of DM, gender, age, PS, clinical stage, histology, smoking history, body mass index (BMI), laboratory parameters, radiation dose, and chemotherapy regimens were obtained retrospectively from the medical charts. Some variables were divided into categories: gender (male or female), PS (0 or 1), clinical stage (IIIA or IIIB), histology (adenocarcinoma or non-adenocarcinoma, squamous cell carcinoma or non-squamous cell carcinoma), smoking history (never or former/current) and DM (present or absent) at the start of CRT. Patients were identified as having DM on the basis of an elevated fasting glucose level (>126 mg/dL), and a history of DM or medication use, such as insulin or oral hypoglycemic agents. All patients underwent systematic evaluation and standardized staging procedures before the start of treatment. Clinical stage was assigned based on the results of physical examination, chest radiography, computed tomography (CT) scans of the chest and abdomen, CT or magnetic resonance imaging (MRI) of the brain, and bone scintigraphy or positron emission tomography (PET). Patients with distant or contralateral hilar lymph node metastases were excluded from this analysis. The histologic classification of the tumor was based on the criteria of the World Health Organization [28]. Our study is conducted according to STROBE guidelines, and we ensure this statement. And this study has been approved by the institutional review board of Shizuoka cancer center (ethical committee for clinical studies- Shizuoka cancer center) and has therefore been performed in accordance with the ethical standards laid down in the 1964 Declaration of Helsinki and its later amendments.

### Treatment methods

Radiotherapy was administered using 6- or 10-MV X-rays in 2-Gy fractions 5 times weekly. All patient treatment plans were designed based on a 3-dimensional treatment planning system. The gross tumor volume was delineated according to nodal involvement determined by CT. The clinical target volume was defined and contoured with 5–10 mm around the gross tumor volume and contours around the regional lymph node regions, i.e., the ipsilateral hilum and the mediastinum. Planning target volume (PTV) 1 comprised the clinical target volume plus a 5 to 10 mm margin; PTV 2 included the gross tumor volume plus a 10 mm margin. An additional margin was added if necessary. Beam shaping was performed using a multileaf collimator. The standard of practice was to prescribe 60 Gy to PTV 2 and 40 Gy to PTV 1. Other objectives were to restrict the relative volume of the normal lung exposed to a radiation dose >20 Gy (V20) to ≤35 %, and the maximum spinal cord dose was restricted to <44 Gy. The dose was prescribed to the isocenter of this point. The chemotherapy regimen was determined by the treating physician.

### Assessment of outcomes and statistical analysis

Radiographic tumor responses were evaluated according to the Response Evaluation Criteria in Solid Tumors, ver. 1.1 [29]. All of the analyses were performed using the JMP statistical software program package (JMP version 9.0 for windows). To explore prognostic factors for Overall survival (OS), the proportional hazards model with a stepwise regression procedure was applied. Hazard ratios (HR) and 95 % confidence intervals (CIs) were estimated using the model. Because the HR is defined for a 1-unit difference, some factors were converted to an appropriate scale unit. Progression-free survival (PFS) was calculated from the start of treatment to the date of PD or death from any cause. OS was calculated from the start of the first cycle of chemotherapy to the date of death from any cause or the date of the last follow-up, and was estimated using the Kaplan-Meier method. The Fisher’s exact test and Wilcoxon rank sum tests were used to compare the mean values of the variables of the two groups studied. The Cox proportional hazards regression model was used to determine the statistical significant of the variables related to survival. Differences were assumed to be significant when the *p* value was less than 0.05.

## Results

### Patient characteristics

**Fig. 1:**
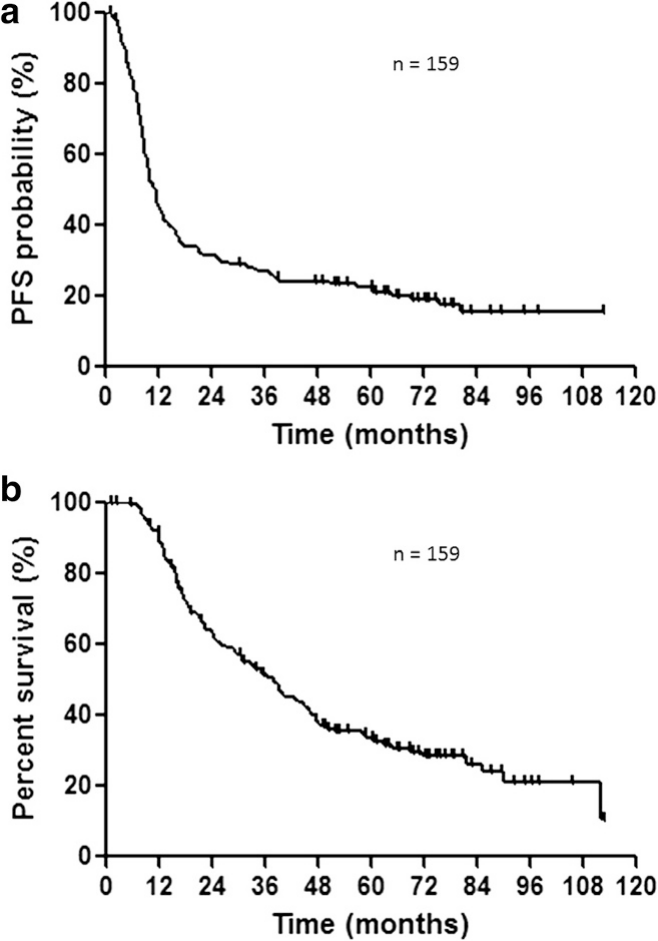
**a** Kaplan-Meier plots showing progression-free survival (PFS). Median PFS: 11.6 months. **b** Kaplan-Meier plots showing overall survival (OS). Median OS: 38.0 months

One hundred and fifty-nine patients with clinical stage III NSCLC were enrolled in this study. The patients’ baseline characteristics are listed in Table 1. There were 30 patients with DM and 129 without DM. The median age of patients was 64 years (range, 40–75 years), with 126 (79.2 %) men and 33 (20.8 %) women. Eighty-six patients (54.1 %) were diagnosed as having stage IIIA and 73 patients (45.9 %) stage IIIB disease. As shown in Table 2, 6, 107, 39 and 5 of all 159 patients showed complete response (CR), partial response (PR), stable disease (SD), and progressive disease (PD), respectively. The response rate was 71.1 % and the disease control rate was 96.8 %. The estimated median PFS was 11.6 months and the median OS was 38.0 months (Fig. 1a and b).

**Table 1:** Patient characteristics

Characteristic		Number of patients
Diabetes Mellitus	Yes	30
	No	129
Gender	Male	126
	Female	33
Age (years), median (range)		64 (40–75)
Performance status	0	90
	1	67
	2	2
Clinical Stage	IIIA	86
	IIIB	73
Histology	Adenocarcinoma	87
	Squamous cell carcinoma	54
	Large cell carcinoma	6
	Others	12
Smoking history	Current or former	119
	Never	25
	Unknown	15
Body mass index (BMI) (kg/m^2^)	BMI < 18.5	15
	18.5 ≤ BMI < 25	116
	25 ≤ BMI < 30	26
	BMI ≥ 30	2
Laboratory parameters, median		
	White blood cell, cells/μl	7070
	Hemoglobin, g/l	13.5
	Albumin, g/dl	4.0
	AST, U/l	20
	ALT, U/l	19
	LDH, U/l	191
	Calcium, mg/dl	9.0
	Creatinine, mg/dl	0.71
	Glucose, mg/dl	102.5
Median (range) radiation dosage (Gy)		60 (46–74)
Chemotherapy regimen	CDDP + VNR	44
	CDDP + S1	46
	CBDCA + PTX	46
	Others	23

*AST* Alanine aminotransferase, *AST* Aspartate aminotransferase, *LDH* Lactate dehydrogenase, *CDDP* Cisplatin, *VNR* Vinorelbine, *CBDCA* Carboplatin, *PTX* Paclitaxel

**Table 2:** Response to chemoradiotherapy

	Number of patients (%)
CR	6 (3.8)
PR	107 (67.3)
SD	39 (24.5)
PD	5 (3.2)
Response rate (%)	71.1
Disease control rate^a^ (%)	96.8

*CR* Complete response, *PR* Partial response, *SD* Stable disease, *PD* Progressive disease

^a^CR + PR + SD

### Prognostic factor analysis

The results of univariate analysis are summarized in Table 3. Among the 16 factors analyzed, five were identified as having prognostic significance: stage (*p* < 0.001), DM (*p* = 0.04), hemoglobin levels (*p* = 0.003), serum albumin (*p* <0.001) and lactate dehydrogenase (LDH) levels (*p* = 0.01). OS was longer amongst patients without DM than with DM (median, 40.3 versus 36.4 months; HR, 1.66; 95 % CI, 1.01–2.63; *p* = 0.031) (Table 4). Furthermore, among the factors tested using Fisher's exact test and Wilcoxon’s rank sum test, two were found to have prognostic significance: gender (*p* = 0.019) and plasma glucose level (*p* <0.001) (Table 5).

**Table 3:** Univariate Cox regression analysis of baseline patient characteristics

	Overall survival		
Factors	Hazard ratio	95 % CI	*p* value
Gender			
Male/Female	1.2	0.80–2.11	0.30
Age	0.99	0.97–1.02	0.75
Performance status			
0/1	1.24	0.84–1.83	0.26
Clinical stage			
IIIA/IIIB	1.92	1.30–2.85	**<0.001**
Histology			
Ad/Non-ad	0.74	0.50–1.09	0.13
Sq/Non-sq	1.19	0.79–1.76	0.37
Smoking history			
Yes/No	1.39	0.82–2.25	0.19
Diabetes Mellitus			
Yes/No	1.66	1.01–2.63	**0.04**
Body mass index (BMI) (kg/m^2^)			
BMI < 18.5 / 18.5 ≤ BMI < 25	1.54	0.77–2.78	0.20
25 ≤ BMI < 30 + BMI ≥ 30/18.5 ≤ BMI < 25	0.95	0.55–1.55	0.85
Laboratory parameters			
White blood cell count	1	0.99–1.00	0.63
Hemoglobin	0.85	0.76–0.94	**0.003**
Albumin	0.4	0.26–0.62	**<0.001**
AST	1.01	0.99–1.03	0.22
ALT	1	0.99–1.02	0.33
LDH	1	1.00–1.05	**0.01**
Calcium	0.81	0.49–1.35	0.43
Creatinine	1.24	0.37–3.88	0.71
Glucose	0.99	0.98–1.00	0.37

*95 % CI* 95 % confidence interval, *Ad* Adenocarcinoma, *Sq* Squamous cell carcinoma, *AST* Alanine aminotransferase, *AST* Aspartate aminotransferase, *LDH* Lactate dehydrogenase, Boldfaced *p*-values are statistically significant (*p* < 0.05)

**Table 4:** Association between OS and DM from univariate analysis

	Median OS (months)	Hazard ratio	95 % CI	*p* value
DM (−)	40.3	1.66	1.01–2.63	0.03
DM (+)	36.4			

*OS* Overall survival, *95 % CI* 95 % confidence interval, *DM* Diabetes mellitus

**Table 5:** Association between DM and other prognostic factors from univariate analysis

		DM (−)	DM (+)	*p* value
Gender	Male	98	28	**0.01** ^**a**^
	Female	31	2	
Age, median (range)		64 (40–75)	64.5 (49–74)	0.44^b^
Performance status	0	72	18	0.51^a^
	1	56	11	
	2	1	1	
Stage	IIIA	70	16	0.92^a^
	IIIB	59	14	
Histology	Adenocarcinoma	71	16	0.76^a^
	Squamous cell carcinoma	45	9	
	Large cell carcinoma	4	2	
	Others	9	3	
Smoking history	Current or former	95	24	0.22^a^
	Never	23	2	
	Unknown	11	4	
Body mass index (BMI) (kg/m^2^)	BMI < 18.5	13	2	0.69^a^
	18.5 ≤ BMI <25	94	22	
	25 ≤ BMI < 30	20	6	
	BMI ≥ 30	2	0	
Laboratory parameters, median				b
	White blood cell, cells/μl	7060 (2950–22310)	7275 (4290–13580)	0.67
	Hemoglobin, g/l	13.4 (8.7–17.2)	13.6 (7.2–16.0)	0.49
	Albumin, g/dl	4.1 (2.4–4.8)	4.0 (3.2–4.9)	0.71
	AST, U/l	20 (11–60)	20.5 (11–50)	0.32
	ALT, U/l	18 (5–67)	22.5 (9–63)	0.16
	LDH, U/l	187 (121–591)	208 (135–1120)	0.05
	Calcium, mg/dl	9.0 (7.6–10.6)	9.05 (8.2–9.9)	0.59
	Creatinine, mg/dl	0.71 (0.4–1.28)	0.73 (0.48–1.23)	0.43
	Glucose, mg/dl	100 (62–144)	118 (85–230)	**<0.001**

*DM* Diabetes mellitus, *AST* Alanine aminotransferase, *AST* Aspartate aminotransferase, *LDH* Lactate dehydrogenase

^a^Fisher's exact test; ^b^Wilcoxon rank sum test; Boldfaced *p*-values are statistically significant (*p* < 0.05)

Multivariate analysis included the five factors identified as significant prognostic predictors by univariate analysis (Table 6), and revealed that stage, DM, and serum albumin and LDH levels were independent prognostic factors for survival (*p* = 0.007, *p* = 0.024, *p* = 0.007 and *p* = 0.005, respectively).

**Table 6:** Multivariate Cox regression analysis for gender, stage, presence of diabetes mellitus, hemoglobin, serum albumin level, serum LDH level and plasma glucose level

	Overall survival		
Factors	Hazard ratio	95 % CI	*p* value
Gender	1.16	0.67–2.07	0.584
Stage	1.75	1.16–2.65	**0.007**
Diabetes mellitus	1.91	1.09–3.23	**0.024**
Hemoglobin	0.94	0.81–1.09	0.444
Albumin	0.46	0.26–0.81	**0.007**
LDH	1.00	1.00–1.01	**0.005**
Glucose	0.99	0.98–1.00	0.063

*95 % CI* 95 % confidence interval, *LDH* Lactate dehydrogenase, Boldfaced *p*-values are statistically significant (*p* < 0.05)

## Discussion

In this retrospective study, we sought to clarify the impact of DM on the prognosis of patients with locally advanced NSCLC, and in particular the relationship between DM and the OS of patients receiving chemoradiotherapy. The prevalence of DM and cancer are both increasing. In our study, the prevalence of DM amongst locally advanced NSCLC patients was 18.9 %, about four times as high as the global prevalence of diabetes [30]. DM (mainly type 2 diabetes) is associated with an increased risk of diverse cancers including those of the liver, pancreas, endometrium, colon, rectum, breast, and bladder [31]. Moreover, preexisting diabetes in cancer patients at the time of diagnosis was associated with an HR of 1.41 for the risk of all-cause mortality compared to patients without diabetes in a pooled analysis of various types of cancer. For instance, preexisting diabetes was significantly associated with increased long-term, all-cause mortality for cancers of the endometrium (HR, 1.76; 95 % CI, 1.34–2.31), breast (HR, 1.61; 95 % CI, 1.46–1.78), and colorectum (HR, 1.32; 95 % CI, 1.24–1.41) [32]. DM is generally characterized by insulin resistance and hyperinsulinemia [33], and insulin resistance is among the hallmark conditions that characterize type 2 DM and leads to hyperinsulinemia.

It has been reported that cancer patients with DM have a poorer short- and long-term prognosis compared to those without DM [32, 34]. However, only a few studies have addressed the impact of DM on the survival of lung cancer patients [20–27]. The only available evidence is limited and conflicting. Although several reports suggested a shorter survival for patients with DM compared to those without DM [20–22], several others have shown that DM was associated with equal or even prolonged survival [23–27]. These studies included patients with metastatic NSCLC, and to our knowledge, the present study is the first to evaluate the relationship between DM and prognostic significance in a sample limited to patients with locally advanced NSCLC who underwent chemoradiotherapy.

The current study demonstrated that patients with DM (*n* = 30) exhibited a significant increase in mortality compared with those without DM (*n* = 129), and that DM is an independent risk factor for survival. There may be several reasons for this. First, insulin activates the phosphoinositide 3-kinase (PI3K)/Akt and the Ras/MAP kinase pathways via the insulin and IGF-1 receptors. These pathways have been shown to stimulate cell proliferation, metastasis and progression. Also, PI3K/Akt pathway plays an important role in treatment resistance, radioresistance and chemoresistance. PI3K/Akt pathway is associated with three major radiation mechanisms (intrinsic radiosensitivity, proliferation and hypoxia). While pAkt has been studied as prognostic factor in NSCLC, inhibition of pAkt increased apoptosis and improved cellular responsiveness to chemotherapy and irradiation in NSCLC. The combination of EGFR inhibitor and PI3K/Akt showed additive cytotoxicity [35, 36]. Insulin resistance is a characteristic of type 2 DM and leads to hyperinsulinemia. Excessive insulin action associated with insulin resistance is thought to progress these multiple cancer phenotypes. Hyperinsulinemia may also promote carcinogenesis indirectly through its effects on IGF-1, as insulin inhibits IGF-1 binding proteins and thus increases the bioavailability of IGF-1. Second, hyperglycemia increases superoxide production by inducing mitochondrial dysfunction. Increased oxidative stress plays a pivotal role in the development micro and macrovascular complications, and also increases the likelihood of DNA damage, leading to an increased rate of mutations and changes in cancer related gene expression. Since cancer cells are anaerobic in their metabolism, and thus require relatively large amounts of glucose, the elevated glucose levels in DM can promote cancer cell survival under hypoxic conditions. Third, chronic inflammation is related to both obesity and DM, and inflammation plays a role in every stage of tumorigenesis, from initiation through to metastatic progression. DM-related inflammation is promoted by the activation of a number of signaling pathways, including those dependent on interleukin-6, tumor necrosis factor (TNF-a), NF kappa-B (NF-kB), STAT3 and adipokines [31, 37–39]. Especially, STAT3 has been studied as prognostic factor in NSCLC. STAT3 related to the sensitivity to cytotoxic agents. In NSCLC cell line, overexpression of STAT3 mRNA levels showed cisplatin-resistance. By contrast, silenceing STAT3 demonstrated more sensitive to the cytotoxic agents. Also, STAT3 plays a crucial role in chemotherapy [40].

The findings of a previous study suggested that DM at the time of diagnosis had no association with survival in patients with SCLC [41], although small cell lung cancer (SCLC) patients generally only survive for a short time anyway, and hence survival may not be affected by the presence of DM. In contrast, DM was found to be a negative prognostic factor for the treatment of advanced NSCLC [22], although at least half the patients in that study had stage IV disease, and thus also had a poor prognosis. In our study, we only included patients who had stage III disease and who were treated with CRT, and the median OS of this cohort was 38.0 months. As these patients were in sufficiently good health to undergo CRT, they correspondingly survived longer on average than patients with stage IV disease. DM also contributes to the longer-term outcome of tumors, and the prognosis after CRT may be affected by relatively long-term insulin resistance and hyperinsulinemia, hyperglycemia, and chronic inflammation associated with this condition. However, further research is needed to clarify how DM affects the outcome after chemotherapy in various types of human cancers.

Previous studies have compared the cancer risk among different ethnic groups, and this revealed that Asian men had a higher cancer risk than non-Asian men. In this context, it is also noteworthy that preexisting DM has a significant impact on NSCLC in Asian men　[42]. Moreover, it was reported that the stage at initial diagnosis, and serum albumin and LDH levels were significant prognostic factors in NSCLC [9, 10, 12–14]. These findings are generally in agreement with those of our study.

This study has several limitations. First, it was a retrospective analysis. Therefore, to minimize biases, all consecutive patients treated within our institutes were included in the analyses, and the patients’ original charts were thoroughly reviewed. Second, we did not evaluate the type of DM, duration of diabetes and the types of diabetic therapy used. Third, our findings may have a selection bias because of the possibility of undiagnosed DM among patients classified as not having DM.

## Conclusions

In conclusion, the presence of DM at the time of diagnosis was identified as an independent and significant prognostic factor for worse survival in locally advanced NSCLC patients. Our findings also suggest that the presence of DM can help predict survival after CRT and is useful for selecting the most appropriate treatment. Therefore, a prospective large-scale trial is warranted to confirm the results of our study.

## Abbreviations

NSCLC: Non-small cell lung cancer
DM: Diabetes mellitus
LDH: Lactate dehydrogenase
CRT: Chemoradiotherapy
PS: Performance status
ECOG: Eastern Cooperative Oncology Group
BMI: Body mass index
CT: Computed tomography
MRI: Magnetic resonance imaging
PET: Positron emission tomography
PTV: Planning target volume
PFS: Progression-free survival
OS: Overall survival
HR: Hazard ratios
CI: Confidence interval
CR: Complete response
PR: Partial response
SD: Stable disease
PD: Progressive disease
MAP: Mitogen-activated protein
IGF-1: Insulin-like growth factor-1
EGFR: Epidermal growth factor receptor
TNF-a: Tumor necrosis factor-a
NF-kB: Nuclear factor-kappa B
STAT3: Signal transducers and activator of transcription 3
SCLC: Small cell lung cancer
AST: Alanine aminotransferase
AST: Aspartate aminotransferase
CDDP: Cisplatin
VNR: Vinorelbine
CBDCA: Carboplatin
PTX: Paclitaxel
Ad: Adenocarcinoma
Sq: Squamous cell carcinoma
